# Knowledge, attitudes, and perceptions of autism spectrum disorder in a stratified sampling of preschool teachers in China

**DOI:** 10.1186/s12888-016-0845-2

**Published:** 2016-05-13

**Authors:** Yingna Liu, Jialing Li, Qiaolan Zheng, Charles M. Zaroff, Brian J. Hall, Xiuhong Li, Yuantao Hao

**Affiliations:** School of Public Health, Department of Medical Statistics and Epidemiology, Sun Yat-Sen University, Guangzhou, China; Faculty of Social Sciences, Department of Psychology, The University of Macau, Avenida da Universidade, Taipa, Macau (SAR), People’s Republic of China; Department of Health, Behavior and Society, Johns Hopkins Bloomberg School of Public Health, Baltimore, MD USA; School of Public Health, Faculty of Maternal and Child Health, Preventive Medicine Institute, Sun Yat-Sen University, Guangzhou, China

**Keywords:** Autism, Autism spectrum disorder, China, Chinese, Teacher, Preschool

## Abstract

**Background:**

In China, children with Autism Spectrum Disorder (ASD) can potentially benefit from universal education policies and recent initiatives designed to address the needs of children with developmental disorders. However, adequate schooling is often unavailable for children with ASD, in part because teachers lack the knowledge and skills needed to work with this population. To better understand the current state of knowledge of ASD in China, we surveyed knowledge and attitudes regarding the disorder in preschool teachers.

**Methods:**

A total of 471 preschool teachers in the cities of Guangzhou and Foshan, China completed questionnaires assessing participant demographics, knowledge of typical child development and knowledge of ASD, attitudes towards ASD, practices and self-perceptions of efficacy in the education of children with ASD, and awareness of organizations and intervention approaches devoted to the care of individuals with ASD. The correlation between individual- and school-level variables with current knowledge of typical child development and ASD was examined using univariate and multivariate analyses.

**Results:**

The majority (84 %) of participants answered correctly more than half of the questionnaire items assessing understanding of typical child development. In contrast, 83 % provided inaccurate responses to more than half of the questionnaire items assessing knowledge of ASD. Knowledge of typical child development and knowledge of ASD were both associated with geographic region (teachers in Guangzhou had greater knowledge than those in Foshan, *p* < 0.0001). Knowledge of ASD was also associated with a higher education level (*p* < 0.05) and school type (*p* = 0.023). In general, participants believed fairly strongly in the need for greater service provision for children with ASD, and were receptive towards receiving additional specialized training. Most participants were unaware of ASD-specific organizations and empirically validated intervention approaches.

**Conclusions:**

Knowledge of ASD is lacking in preschool teachers in China, and greater teacher training and instruction is needed. Nonetheless, teachers report a willingness and motivation to gain the skills needed to maximize the educational experiences of children with ASD.

**Electronic supplementary material:**

The online version of this article (doi:10.1186/s12888-016-0845-2) contains supplementary material, which is available to authorized users.

## Background

Autism Spectrum Disorder (ASD) is a neurodevelopmental disorder characterized by persistent deficits in social communication and social interaction, and restricted, repetitive patterns of behavior, interests, or activities [1]. The disorder has a global prevalence of 0.62 % [2], while recent prevalence estimates in the US run as high as 1.4 % [3]. ASD is associated with enormous financial burdens [4], to a far greater extent than that which occurs with other childhood disabilities [5]. However, interventions are available, and can be quite efficacious when applied early in development, leading to improved social communication and social interaction, and even increased IQ scores [6–9]. Prompt intervention is crucial since intensive behavioral interventions may have diminished effectiveness in older children [10, 11].

The implementation of intervention in young children rests upon disorder identification. As such, diagnosis at younger ages plays a crucial role in prognosis and developmental outcomes. Several trends have aided the diagnostic process. While much of the recent increase in prevalence has been attributed to a broadening diagnostic concept and increased ascertainment methods [12, 13], greater awareness of ASD in the general population has also played a contributory role [14, 15], and has been correlated with a younger age at diagnosis [16].

However, this increase in awareness is not universal. In China, while increased understanding of ASD is found among service providers [17], the general population exhibits common inaccurate perceptions concerning disorder etiology and intervention options [18]. Problematic behavior associated with ASD is often attributed to willful disobedience or a disordered personality in China [19]. It is likely that such attributions contribute to delays in help-seeking behavior [18], and are partly responsible for the considerable stigma experienced by families of children with ASD. The relatively low ASD prevalence estimates in China (i.e., 0.11 %) [2, 20] suggest a deficiency in diagnosis, possibly due in part to low levels of awareness in the general population.

In sum, these results highlight the urgent need for increased awareness of ASD in China. One segment of society in which this need may be the most acute is teachers. Global policy and Chinese initiatives give great importance to the instruction of children with special needs within regular classrooms [21–23]. Thus, outside of parents and caregivers, teachers are the group most likely to work the closest with children and adolescents with ASD. Teachers have been the focus of much recent research concerning knowledge of and attitudes towards ASD [24–26]. However, much of this research has been gathered in primary and secondary school instructors [23–27], whereas relatively less attention has been devoted to the state of knowledge in preschool teachers [28, 29], and overall, little work of its kind has been conducted in China. Not only are increasing numbers of children with ASD being enrolled in mainstream preschool programs [30], but educational programs for children with ASD are often most beneficial when initiated at a younger age [31]. Preschool teachers work with the very population in which timely diagnosis is most likely to lead to a beneficial outcome [32]. As such, their ability to detect developmental anomalies and subsequently provide recommendations to parents and school staff for referral to medical care are invaluable.

The study of preschool teacher knowledge and attitudes may further help contribute to a growing body of literature concerning early intervention efforts [28]. Such research may also serve to delineate the impact of policy initiatives in China, which have begun to help address the needs of children with such disorders via expanded financial assistance and more accurate disorder classifications [19]. In the current study, we have adapted survey methods from a study by Lian et al. [28] that assessed preschool teacher knowledge towards ASD in Singapore with psychometric properties validated in pilot studies. One prior pilot study utilized general practitioners [33], and generally found greater knowledge of typical child development relative to knowledge of atypical development manifested in disorders such as ASD and attentional disorders, knowledge that was not associated with either age or gender of the participants. Our research efforts were directed as follows:

1. Assess the knowledge preschool teachers possess regarding typical child development and ASD.
2. Assess individual- and school-level variables associated with an accurate understanding of typical child development and ASD.
3. Assess the attitudes teachers hold toward the needs of children with ASD, and perceptions concerning their own potential role and ability in working with children with ASD.
4. Assess teacher awareness concerning organizations and intervention approaches devoted to the care of individuals with ASD.

## Methods

### Participants

Ethical approval was obtained by the School of Public Health at Sun Yat-Sen University. Informed consent was obtained prior to study participation. Teachers were free to participate and were informed that a refusal to participate would in no way affect their work status within the school. A total of 471 teachers completed the survey for analysis; out of the total number of potential participants, this represents a 97 % response rate.

### Assessment measures

All questionnaires and individual questionnaire items were adapted for a Chinese cultural context from measures utilized in prior studies assessing awareness of ASD. The questionnaire assessing knowledge of ASD utilized item content from prior studies [18, 28, 34] created for this purpose, and these prior studies generally utilized pilot studies to ensure item clarity and adequate psychometric properties [18, 28, 33].

The questionnaires (Additional files 1 and 2) assessed 1) knowledge of typical child development, 2) attitudes pertaining to the needs of children with ASD, 3) interest and perceived efficacy in working with children with ASD, and, 4) knowledge of organizations and intervention approaches devoted to individuals with ASD, and were adapted from a study of preschool teachers of predominantly Chinese ethnicity in Singapore conducted by Lian et al. [28]. Lian and colleagues designed questionnaire items and conducted a pilot study in a sample of general practitioners [33]. They subsequently refined the measures on the basis of feedback from this pilot study, before utilizing the measures in a second study [28].

### Participant demographics

Participants completed a questionnaire assessing their age, gender, educational experience, prior experience working with children with special needs, and current school type (Province-, City-, or District-level). Schools are designated types depending on a comparison of factors against a standard, including the number of students per class, area of the school, salary of teachers, and more [35]. Among the factors, Province-level schools must achieve 85 % at the highest rating, City-level schools must achieve 75 %, and District-level schools must achieve 60 % [35]. For example, Province-level schools must provide a greater quality of education of students, such as having fewer students per class and a greater estate area than City- or District-level schools. Consequently, the funding of school types would vary with schools at the Province-level receive higher levels of funding than those at the City-level, which in turn receive greater amounts of funding than those at the District-level.

### Knowledge of typical child development

A questionnaire consisting of 14 true/false statements (with an option for ‘I do not know’) about typical child development from the study by Lian et al. [28] was utilized. Sample items included, ‘It is normal for a one and a half year-old-child to have already developed a definite hand preference’ [correct response = false] and, ‘All children with speech and language delay should have a hearing test’ [correct response = true]. One point was awarded for each correct response, whereas omitted responses and those answered ‘I do not know’ received no credit. Following the methodology utilized by Lian and colleagues [28], participants were designated as having ‘passed’ the measure if they provided correct responses to more than 50 % of the items (i.e., >7/14).

### Knowledge of ASD

A questionnaire consisting of 17 true/false statements (with an option for ‘I do not know’) about ASD was administered. Items were derived from prior studies conducted for this purpose [18, 28, 34]. Sample items included, ‘Autism is a developmental disorder’ [correct response = true] and, ‘Children with autism usually grow up to be adults with schizophrenia’ [correct response = false]. One point was awarded for each correct response, whereas omitted responses and those answered, ‘I do not know’ received no credit. Following the methodology utilized by Lian and colleagues, participants were designated as having ‘passed’ the measure if they provided correct responses to more than 50 % of the items. The final two items, as they were used to query participant beliefs concerning the use of Traditional Chinese Medicine (TCM) in the treatment of ASD to assess the influence of TCM in the understanding of ASD, were omitted from this scoring system.

### Attitudes towards the care and education of, and advocacy for, children with ASD

A ten-item questionnaire of attitudes and perceptions was adapted from the survey by Lian et al. [28]. Questionnaire items contained a 5-point Likert scale response system, from 1 (‘strongly disagree’) to 5 (‘strongly agree’). Individual items assessed attitudes concerning entitlements for children with ASD (e.g., ‘Preschools should have special education teachers and therapists to provide services for children with special needs’; ‘Parents are responsible for obtaining services for their own children with special needs’). Likert-style responses were averaged for each questionnaire item.

### Interest and perceived efficacy

An eight-item questionnaire measuring interest and self-perceived efficacy in working with children with ASD was adapted from the study by Lian et al. [28]. Each response was completed on a 5-point Likert scale ranging from 1 (‘strongly disagree’) to 5 (‘strongly agree’). Sample items included, ‘I feel equipped to handle children with special needs,’ and, ‘I want to make a difference in the education of children with special needs.’ Likert-style responses were averaged for each questionnaire item.

### Awareness of organizations devoted to the care of individuals with ASD

A five-item questionnaire was created, assessing awareness of organizations and institutions devoted to the care of individuals with ASD in China. This survey was based on a similar questionnaire designed by Lian et al. [28]. In the current study, the institutions chosen for inclusion were specific to those in China, and were chosen for their prominence, and geographic proximity to the schools surveyed. For example, Beijing Stars and Rain provides intensive parent-training, residential facilities, and a preschool program for children with ASD, utilizing principles of applied behavior analysis. While located in Beijing, it has been featured in international media and identified by the Ministry of Education in China as an exemplary institute [36]. In contrast, the Kangna School, the first government-run facility in Guangdong province for individuals with ASD, was chosen specifically for its prominence, but also for its proximity to the schools participating in the current study. Similarly, other regional associations, generally privately-funded and attended (e.g., Shenzhen Autism Association; Guangzhou TaiYangChuan Rehabilitation and Education Centre), were also chosen for inclusion. For individual questionnaire items, the number of ‘yes’ responses were summed, and percentages of ‘yes’ responses for each item were calculated.

### Awareness of intervention approaches devoted to the care of individuals with ASD

A five-item questionnaire was created, assessing awareness of intervention approaches for use in ASD. This list was partially devised from expert consensus on best practices, such as applied behavior analysis [37], and also from a literature search of practices most commonly utilized in China, such as sensory integration treatment [36]. The number of ‘yes’ responses was summed, and the percentages of ‘yes’ responses for each item were calculated.

### Procedures

In order to assess legibility, and ensure clarity of translation, pilot data were gathered on the questionnaires in a sample of 26 teachers at a single school in Guangzhou in January of 2013. Feedback from this pilot study concerning item wording was integrated into the final version of the questionnaire items. The questionnaires were then administered to teachers at 21 preschools in the cities of Guangzhou and Foshan over the span of eight weeks (April of 2013 to June of 2013). These regions were chosen in order to provide comparison across urban areas situated more internationally, and those situated more regionally. Guangzhou is the capital of Guangdong province, and China’s third most populous city. Multiple globally ranked institutions of higher learning are situated in this region, suggesting a proximity to centers of research and learning. Thus, in China, when outreach programs provide information about specific instructional methodologies for children with ASD, the common targets of such programs are regions such as Guangzhou [38]. In contrast, Foshan is a smaller city lacking access to such services and resources. For example, while the Gross Domestic Product of Guangzhou ranks only behind Shanghai and Beijing, and in terms of recent growth, outpaces both of the aforementioned cities [39], the Gross Domestic Product of Foshan ranks third within Guangdong province itself [40].

Guangzhou consists of 10 separate school districts from which regional governmental permission was solicited. This resulted in the approval for the study of two school districts: Yuexiu District Bureau of Education and Haizhu District Bureau of Education. In Foshan, schools affiliated with the Jiujiang District Bureau of Education participated. Schools were selected via stratified sampling utilizing school-level variables (i.e., Province-level, City-level, District-level).

Research assistants reviewed completed questionnaires for item omissions. In the case of item omissions, research assistants returned the questionnaires to teachers with a reminder. Nonetheless, participation in the study was entirely voluntary and an omission of responses was permitted.

### Statistical analyses

Descriptive statistics were derived for each questionnaire. Following criteria set forth by Lian and colleagues [28], participants were considered to have ‘passed’ the knowledge tests when achieving scores greater than 50 %. Univariate analyses (i.e., *t-*tests or Analysis of Variance; ANOVA) were utilized to contrast knowledge scores on the basis of demographic variables. A multiple linear regression was conducted to explore the contributions of demographic variables to both knowledge tests, and a score comprised of the sum of performance on the two individual knowledge tests. Significance level was at 0.05 for all analyses. All analyses were carried out using the Statistical Package for the Social Sciences (SPSS) version 17.0.

## Results

### Participant demographics

We invited 483 teachers from Guangzhou and Foshan for participation. We surveyed 474 teachers with 100 % participation but discarded 3 teacher responses due to lack of completion. A total of 471 teachers completed the survey for analysis (Table 1), the overwhelming majority of whom were female. Teachers had an average of 9.89 + 8.14 years of work experience. Teachers generally reported (83 %) receiving formal training in early childhood development. In contrast, while more than half of the sample reported having had work experience with children with special needs, only 16 % had received training to do so.

**Table 1:** Participant demographic data

	Number	Percent
Gender		
Male	4	<1
Female	467	99 %
Age in years		
<20	21	4.4
20–24	103	21.9
25–29	82	17.4
30–34	90	19.1
35–39	69	14.6
40–44	61	12.9
45–59	23	4.8
≥50	21	4.4
Education		
≤ Middle school	6	1.2
High school	23	4.9
Middle vocational	86	18.4
High vocational	220	47.1
College	132	28.2
≥ Masters	0	0
Years teaching preschool (median)	9	
Formal training in early child development	391	83 %
Formal training in instruction of children with special needs	76	16.3 %
Work experience with children with special needs	270	58.3 %
School type		
District	168	35.6
City	163	34.6
Province	140	29.7

### Knowledge of typical child development/Knowledge of ASD

On the questionnaire assessing knowledge of typical child development, the average score was 9.49 ± 1.95 (out of a possible total of 14 points), and 84 % of the sample answered more than 50 % of the items accurately (items answered correctly by 50 % or less of the sample are provided in Table 2). In contrast, on the questionnaire assessing knowledge of ASD, the average score was 5.53 ± 2.06 (out of a possible total of 15 points), and only 17 % of the sample answered more than 50 % of the items accurately (items answered correctly by 50 % or less of the sample are provided in Table 3). The percentage of total correct responses was significantly higher on the questionnaire assessing knowledge of typical child development than that assessing knowledge of ASD (*p* < 0.0001). On the questionnaire assessing knowledge of ASD, several inaccuracies were identified: most teachers attributed the disorder to a psychological problem that was entirely curable if diagnosed and treated at a young age. The last two questions assessing knowledge of ASD inquired about participant beliefs concerning the relationship between TCM and ASD. Half of the participants (50 %) were unsure of the effect of the principles of yin/yang on ASD (43 % did not believe in an effect). In contrast, 70 % agreed that ASD does not manifest as physical pain.

**Table 2:** Knowledge of typical child development questionnaire items failed by ≥ 50 % of the sample

Question Response	Correct	% correct (95 % CI)
There is no cause for concern if a three-year-old child can recognize all Chinese characters, but does not speak in sentences.	False	50 (46–55)
It is normal for a one and a half-year-old child to have already developed definite hand preference.	False	46 (42–51)
A child with poor language skills can appear hyperactive and inattentive.	True	46 (42–51)
All children with speech and language delay should have a hearing test done.	True	42 (38–47)

Note: CI, confidence interval

**Table 3:** Knowledge of Autism Spectrum Disorder questionnaire items failed by ≥ 50 % of the sample

Question Response	Correct	% correct (95 % CI)
A child with autism often does better with visual input than auditory input.	True	38 (34–42)
Children with autism usually grow up to be adults with schizophrenia.	False	32 (28–36)
Changing the diet of a child with autism will make a difference in their outcome.	False	28 (24–32)
Children with autism do not show social attachments, even to parents.	True	23 (19–27)
Autism occurs in less than 10 % of the population	True	20 (16–24)
Autism is curable if diagnosed early and the appropriate intervention is provided.	False	5 (3–7)
Autism is a psychological problem.	False	4 (2–6)
With the proper treatment, most children with autism outgrow autism.	False	4 (2–6)

Note: CI, confidence interval

The influence of demographic variables on the accuracy of knowledge of typical child development, and accuracy of knowledge of ASD is provided in Tables 4 and 5. Accuracy of knowledge of ASD was associated with school-type (i.e., teachers from City-level schools provided the most accurate responses - univariate analysis: *p* = 0.023; multivariate analysis: *p* = 0.031), geographic region (i.e., teachers in Guangzhou possessed more accurate knowledge than teachers in Foshan - univariate/multivariate analyses: *p* < 0.0001), and teacher education level (i.e., teachers with middle vocational educational training or higher had significantly more accurate knowledge - univariate/multivariate analyses: *p* < 0.05).

**Table 4:** Questionnaire performance based on demographic data

	Composite score Maximum = 29 Mean (SD)	Typical child development Maximum = 29 Mean (SD)	Typical child development Maximum = 29 Mean (SD)
Total	15.02 (3.09)	9.49 (1.95)	5.53 (2.06)
Education			
≤ Middle school	12.83 (0.75)	8.83 (1.117)	4.00 (0.89)
High school	14.65 (3.52)	9.09 (1.96)	5.57 (2.35)
Middle vocational	15.08 (3.13)	9.40 (1.98)	5.69 (1.92)
High vocational	15.07 (3.01)	9.58 (1.92)	5.49 (1.99)
College	15.17 (3.09)	9.55 (1.99)	5.61 (2.18)
Formal training in early child development			
Yes	15.14 (3.01)	9.54 (1.89)	5.60 (2.01)
No	14.44 (3.43)	9.23 (2.20)	5.21 (2.25)
Work experience with children with special needs			
Yes	15.27 (3.12)	9.60 (1.99)	5.67 (2.05)
No	14.58 (2.99)	9.27 (1.89)	5.31 (2.04)
School type			
District	15.17 (3.09)	9.54 (1.94)	5.63 (2.10)
City	15.09 (3.13)	9.32 (2.05)	5.77 (1.80)
Province	14.76 (3.06)	9.62 (1.84)	5.14 (2.22)
City			
Guangzhou	15.62 (2.30)	9.73 (1.90)	5.89 (2.06)
Foshan	14.24 (3.05)	9.18 (1.97)	5.07 (1.97)

Note: SD, standard deviation

**Table 5:** Predictors of composite score performance

	B	SE (B)	β	*t*	Sig. (*p*)	95 % CI for B
City	−0.889	0.201	−0.217	−4.420	<.0001	−0.129, −0.49
School type (Province)	−0.542	0.250	−0.122	−0.2168	0.031	−1.03, −0.05
School type (City)	−0.006	0.230	−0.001	−0.026	0.979	−0.46, 0.45
Gender	0.091	1.015	0.004	0.090	0.928	−1.90, 2.09
Age	0.022	0.021	0.099	1.087	0.277	−0.02, 0.06
Education (High school)	1.525	0.942	0.162	1.618	0.106	−0.33, 3.38
Education (Middle vocational)	2.008	0.904	0.387	2.200	0.027	0.23, 3.79
Education (High vocational)	1.913	0.896	0.471	2.136	0.033	0.15, 3.67
Education (College)	1.842	0.909	0.411	2.025	0.043	0.05, 3.63
Teacher experience	−0.029	0.022	−0.119	−1.314	0.189	−0.07, 0.02
Formal training in early child development	−0.244	0.266	−0.045	−0.917	0.359	−0.77, 0.28
Child with special needs in the classroom	−0.0254	0.205	−0.063	−1.243	0.215	−0.66, 0.15
Previous experience with special needs	0.122	0.255	0.023	0.480	0.631	−0.38, 0.62
Awareness of ASD	0.430	0.546	0.038	0.789	0.431	−0.64, 1.50

Note: *F* = 2.532, *p* = 0.02; For School type, District is set as the reference. For Education, Middle school is set as the reference

**Teacher experience =** duration of preschool teaching experience. **Child with special needs in the classroom** = a history of instruction of a class with at least one child with special needs. **Previous experience with special needs** = a history of formal training in the instruction of children with special needs. **Awareness of ASD** = awareness of the term ASD

A composite score was created, consisting of the sum of accurate responses on the questionnaire assessing knowledge of typical child development and that assessing knowledge of ASD. On this composite score, a significantly greater accuracy level was observed in teachers from Guangzhou (univariate/multivariate analyses: *p* < 0.0001), and in teachers with prior work experience with children with special needs (univariate/multivariate analyses: *p* < 0.001). Differences in the accuracy of knowledge of typical child development were also observed, favoring teachers from Guangzhou (univariate/multivariate analyses: *p* = 0 .001). There were non-significant trends for more knowledge about ASD among teachers with prior work experience with children with special needs and among teachers with prior training in early child development.

### Attitudes towards the care and education of, and advocacy for, children with ASD

Attitudes regarding the needs of children with ASD were on average fairly neutral or slightly favored improving education for children with ASD (Table 6). In general, no strong opinions were expressed regarding the potential placement of children with special needs into mainstream schooling environments or regarding the presence of parents of children with special needs in the classroom. However, 72-73 % of teachers felt strongly that increased government funding should be made available for staffing and training needs for teachers working with children with special needs. Comparable percentages of teachers expressed similarly strong sentiments concerning the provision of expanded insurance to cover costs.

**Table 6:** Attitudes towards the care and education of, and advocacy for, children with Autism Spectrum Disorder

Item	Statement	Average score	1	2	3	4	5
1	Children with special needs should be integrated into mainstream school.	3.25	16 %	14 %	27 %	14 %	29 %
2	All preschools should allow children requiring special education to attend their classes while waiting placement.	2.69	24 %	21 %	31 %	9 %	15 %
3	Preschools should allow parents of children with special needs in the classroom.	2.64	33 %	16 %	21 %	14 %	16 %
4	All preschools should have special education teachers and therapists to provide services to children with special needs attending class there.	3.56	14 %	9 %	23 %	14 %	49 %
5	Government funding should be made available to facilitate staff employment in preschools to meet the needs of these children.	4.42	5 %	2 %	9 %	11 %	72 %
6	Parents should help bear the cost of services within the preschool.	3.66	9 %	9 %	26 %	20 %	36 %
7	There is adequate provision of services of children with special needs in China.	2.72	30 %	19 %	20 %	8 %	22 %
8	The government should allocate more resources for the provision of services for children with special needs.	4.44	6 %	0 %	8 %	11 %	73 %
9	Insurance policies should be amended to include coverage for developmental disorders as chronic disabilities.	4.25	4 %	4 %	16 %	13 %	62 %
10	Parents are responsible for obtaining services for their own children with special needs.	4.28	4 %	3 %	15 %	19 %	60 %

Note: 1 = Strongly disagree, 2 = disagree, 3 = neither disagree nor agree, 4 = agree, 5 = strongly agree

### Interest and perceived efficacy

Concerning practices for the treatment of children with ASD (Table 7), participants generally held opinions that were neutral to slightly favored perceptions of their own training and capabilities. They generally expressed stronger opinions concerning their desires to work with children with special needs, relative to their self-perceived efficacy in doing so.

**Table 7:** Interest and perceived efficacy

Item	Statement	Average score	1	2	3	4	5
1	I feel equipped to handle children with special needs.	3.40	9 %	14 %	32 %	18 %	27 %
2	I am interested in attending training in the area of childhood developmental and behavioral disorders.	3.80	6 %	9 %	24 %	22 %	40 %
3	If adequately trained, I am willing to have children with special needs in my class.	4.15	4 %	5 %	16 %	20 %	54 %
4	I am keen to be a partner in the classroom management of children with special needs (e.g., using specific visual aids, monitoring medication effects).	3.70	7 %	9 %	29 %	20 %	36 %
5	I am happy to have parents or therapists sit in as helpers.	3.55	12 %	9 %	23 %	22 %	33 %
6	I see the need to implement changes in the classroom to accommodate a child with special needs.	4.02	3 %	7 %	22 %	20 %	48 %
7	I want to make a difference in the education of children with special needs.	4.04	4 %	5 %	21 %	22 %	48 %
8	I feel I can make a difference in the education of children with special needs.	3.07	14 %	13 %	41 %	15 %	16 %

Note: 1 = Strongly disagree, 2 = disagree, 3 = neither disagree nor agree, 4 = agree, 5 = strongly agree

### Awareness of organizations devoted to the care of individuals with ASD

Most participants (>70 %) were not familiar with any of the organizations devoted to the care of individuals with ASD chosen for inclusion in the current study (Table 8).

**Table 8:** Awareness of organizations devoted to the care of individuals with Autism Spectrum Disorder

Organization	Yes	No
Beijing Stars and Rain	60 (13 %)	411 (87 %)
Autism Speaks	96 (20 %)	375 (80 %)
Kangna School	138 (29 %)	333 (71 %)
Shenzhen Autism Association	113 (24 %)	358 (76 %)
Guangzhou TaiYangChuan Rehabilitation and Education Center	105 (23 %)	355 (77 %)

### Awareness of intervention approaches devoted to the care of individuals with ASD

More than 60 % of respondents had not heard of certain empirically validated intervention approaches for children with ASD (e.g., applied behavior analysis) (Table 9). In contrast, most participants did report awareness of more sensory-based approaches.

**Table 9:** Awareness of intervention approaches devoted to the care of individuals with Autism Spectrum Disorder

Organization	Yes	No
Applied behavior analysis	174 (37 %)	297 (63 %)
Structured training	94 (20 %)	377 (80 %)
Relationship Development Intervention	103 (22 %)	368 (78 %)
Sensory integration therapy	358 (76 %)	113 (24 %)
Auditory integration therapy	336 (71 %)	135 (29 %)

## Discussion

In the current study, in comparison to a strong knowledge base on typical childhood development, knowledge of ASD among preschool teachers in China was lacking. The majority of teachers were unable to provide accurate responses to half of the questionnaire items pertaining to ASD. A conceptualization of ASD as psychological in origin predominated, despite current expert consensus of the disorder as having a strong genetic component [41, 42]. Lack of accurate knowledge of the disorder may in part stem from the Chinese terms for autism, *GuduZheng* or *ZibiZheng*. The literal translations of both terms equate to ‘loneliness disease’ or ‘isolation disease,’ implying a more psychological etiology. Perhaps not surprisingly then, the majority of participants also expressed the view that with proper diagnosis and treatment, the disorder could be ‘cured’ or ‘outgrown.’ These results are especially concerning since 96 % of teachers were familiar with the term ‘ASD,’ which in itself suggests that instruction in early child development is insufficient in providing teachers with a knowledge base in neurodevelopmental disorders.

The lack of graduate level education may have also contributed to the picture captured during the current study. None of the participants had more than a university level education (i.e., none possessed a master’s degree). These results provide a potential explanation for why an undergraduate level of education has been deemed insufficient in providing teachers with the skills needed to work with children with ASD [29]. Supporting such an interpretation is that within the current sample, education level *did* have a significant effect on knowledge of ASD: teachers with vocational school or college instruction scored significantly higher on ASD knowledge tests than teachers with lower levels of academic instruction. However, it is also important to note that on the basis of multivariate analyses, studies have shown that further education in Chinese parents did not significantly affect their knowledge of ASD [18]. Furthermore, consistent with prior research [26, 28], teachers with previous work experience with children with special needs possessed significantly more accurate understanding of ASD (and childhood development overall) than those who did not. Presumably, such face-to-face interactions could increase understanding of child development in general, enabling teachers to be better able to discern differences between typically developing children and those with special needs.

In addition to the influence of teacher background, variables pertaining to the school of instruction also contributed to the accuracy of knowledge of ASD. However, contrary to expectations, teachers at Province-level schools, which have greater resources relative to District-level or City-level schools, possessed on average *less* accurate knowledge of the disorder. The cause of this finding is unclear, although it may reflect the lack of experience working with children with special needs on the part of teachers in Province-level schools. Mandatory education policies for all children, regardless of their ability level, have been required in China [43]. However, in principle, such policies are not enforced. Children with ASD have been refused school enrollment with the rationale that the schools in question lack an understanding and an ability to work with such children [44]. Thus, teachers in the more prestigious state-run schools may be less likely to have had face-to-face experience with children with ASD simply because the parents of such children were forced to turn to less prestigious schools for enrollment [45]. In fact, in Guangdong province, Province-level schools have higher tuition fees than District-level schools, and also utilize quotas in the selection of students. These quotas include families who are able to pay greater fees, students accepted on the basis of results of regional lotteries, and students accepted on the basis of merit. Thus, in Province-levels schools, whereas 10 % of students are government- and/or school-sponsored, up to 60 % of students gain enrollment through a lottery process, owing to the demand for enrollment exceeding available enrollment quotas. In contrast, District-level schools have no such quotas, but rather enroll all local students, with the only preference being given to those residing in greater geographic proximity to the school. It is thus conceivable that Province-level schools may simply enroll a smaller number of students with special needs, particularly since parents with the financial means to do so may choose to enroll their children with special needs in programs dedicated to the education of this population. On the other hand, parents with fewer financial resources may not have the ability to enroll their children in such specialized programs and may instead need to send their children to District-level schools.

Teacher knowledge of ASD (and child development in general) was associated with geographic region. Teachers in Guangzhou had significantly higher knowledge scores than those in Foshan. Guangzhou is the third largest city in China, and it houses several universities. It is probable that teachers in this region are better positioned to learn about and receive training in the education of children with special needs. Research examining the effects of specialized education for children with ASD has been noted to target cities such as Guangzhou [38], although such reports have not made similar mention of Foshan.

Teacher beliefs concerning the interaction of TCM and ASD were assessed with two items. Half of the teachers queried were unsure of whether ASD is caused by a yin/yang imbalance within the body, an idea central to TCM conceptualizations of mental illness [46]. This may suggest that teachers are as likely to refer children with ASD to TCM physicians as physicians from western specialties. On the other hand, 70 % of respondents did not believe that ASD manifests in regional physical pain, another hallmark of TCM. However, this finding may be attributable to the strong belief among respondents that ASD is psychological in nature, thereby negating potential manifestations of physical pain.

An assessment of teacher attitudes towards service provision revealed several trends. While on average, opinions concerning the current state of services were fairly neutral, most participants (>80 %) did believe that expanded funding for children with special needs was needed. These perspectives are in line with views espoused by parents of ASD, who are dissatisfied with services available in China [47]. However, paradoxically, nearly 80 % of participants also expressed the belief that parents, not the school system per se, are responsible for obtaining needed services, and comparable percentages of teachers favored (30 %) and opposed (43 %) an integration of children with special needs into mainstream classrooms. Thus, while participants generally favored expanded government funding and insurance coverage for children with ASD, less agreement was evident concerning the preferred location of such services. This is not surprising given that children with special needs may be refused enrollment in schools in China [19, 48], and even in government-run schools catering to children with special needs, children with ASD may still face difficulty gaining admission [19, 49]. The current results are also potentially problematic in that teachers were more likely to oppose the presence of a parent of a child with ASD in the classroom. However, in the absence of a school support system for children with special needs, parents may advocate for their presence in the classroom [45].

On the other hand, the current results are encouraging in revealing a desire for teachers to participate in the education of children with ASD. In China, the decision to deny enrollment of children with ASD to schools is often attributed to teachers not possessing the knowledge and skills needed to educate this demographic [19]. Thus, while more than 70 % of the current sample expressed a desire to participate in the education of children with special needs, only 31 % felt adequately equipped to do so. Similar findings have been reported elsewhere, as teachers express little confidence in being able to meet the needs of students with ASD [27], but feel that additional instruction would benefit their classroom practice [28]. These findings represent a potentially more positive future for children with ASD in China, who although may receive some funding from the government, they at present have to rely on non-governmental, private organizations [50] or medical institutions for services [51].

We found little awareness of organizations and institutions devoted to the care of individuals with ASD. Eighty-seven percent of teachers were not familiar with Beijing Stars and Rain, a reputable organization for children with ASD that has been publicized in both the lay press and scholarly work [36]. Regional organizations, such as the Kangna School in Guangzhou, fared only a little better (i.e., 29 % were familiar with this school). Regardless of culture, most lay people are exposed to information about ASD via the media [52, 53]. It is thus surprising that this information is lacking, as media coverage in China has tended to increase the advocacy for a charity when discussing ASD [54]. Nonetheless, the total amount of coverage given to ASD in Chinese media is still lacking as compared to that in other geographic regions [54].

Likewise, most teachers did not recognize intervention approaches empirically validated for use in ASD, such as applied behavior analysis. In contrast, most teachers (>70 %) did recognize sensory and auditory integration training. This is not surprising, since relatively few venues are available for applied behavior analysis in China, whereas sensory and auditory integration approaches may be much more commonly utilized [36]. However, the implications of these findings require further research. There has been an acknowledgement by the scientific community of the role sensory deficits/anomalies play in ASD, as evidenced by the decision to include ‘hyper- or hyporeactivity to sensory input’ for the first time as part of DSM diagnostic criteria [1]. However, while at least in the case of auditory integration training, empirical support in western cultures is lacking [55, 56], research in China has shown preliminary evidence of the benefits of more physically-oriented interventions [57]. Regardless, the current findings do corroborate what has been found in samples of parents of children with ASD in China and teachers globally: they lack knowledge concerning intervention approaches and are often not likely to obtain an assessment, much less initiate a treatment [18, 26].

Overall, the current results further highlight how preschool teachers in China tend to be familiar with aspects of typical child development, but have much less familiarity with the nature and interventions validated for atypical child development. In comparison with a sample of teachers in Singapore [28], teachers in the current sample were much better acquainted with facets of typical child development: 84 % were able to answer greater than 50 % of questions on this topic accurately, whereas in Singapore, only 56 % of teachers were able to do so. However, whereas over 60 % of respondents in Singapore were able to provide accurate responses to more than half of the questions administered pertaining to ASD, only 17 % of the current sample were able to do so. Interestingly, in the study by Lian et al. [28], teachers in Singapore were more willing to allow children with special needs into mainstream classrooms and schools. Differences concerning inclusive education across studies may reflect differences in the understanding of the benefits of inclusive education. On the other hand, teachers in both studies did agree on the need for greater governmental responsibility in the expansion of services and insurance coverage for children with special needs.

The current study is not without its limitations. While an attempt was made to compare teachers from a more urban and international region with those from a less urban region, the use of Foshan can be criticized. Foshan neighbors Guangzhou and is likely heavily influenced by its neighboring city. Additional work focused on rural regions is recommended, and may incorporate cities in other regions of China. This need is especially important as our current study found that teachers in less resource-rich schools were more familiar with ASD than those from more resource-rich schools. Thus, there may be an interaction between level of school resources, the likelihood of admission for a student with special needs, and subsequent teacher knowledge. Additionally, while a reasonably large number of teachers were surveyed in the current study, teachers from only 2 of 10 districts in Guangzhou were participants. Population-based studies encompassing more than pre-schools may be more representative of the true state of knowledge of ASD within the community.

## Conclusion

Ultimately, our study corroborates previous findings in China in uncovering a lack of awareness and knowledge concerning ASD in the general population [58]. We found associations between such knowledge and teacher education level and experience as well as geographic location and type of school. Additionally, we found that teachers did in fact recognize their own lack of knowledge of ASD, and that this awareness co-existed with an interest in increasing their knowledge and skills in this area. Lastly, teachers were of the belief that the government should be more involved in the services offered to children with ASD. These results echo calls made elsewhere [59] for the incorporation of programs and educational curricula in teacher-training that focuses on children with special needs. In fact, this topic has come under increasing scrutiny worldwide [28, 59, 60]. It is argued herein that such curricula revisions might have the secondary benefit of adding, in an incremental fashion, to the trend towards earlier diagnoses and intervention, and subsequently, improved outcomes.

### Ethics approval and consent to participate

We received approval from the Institutional Review board of the Sun Yat-Set University School of Public Health, reference number L2016-045, before proceeding with surveying. Each participant was asked for their consent to participate before answering questions.

### Consent to publish

Not applicable.

### Availability of data and materials

All data to support our findings can be found in the manuscript.

## Additional files

Additional file 1: Survey-Chinese.docx: Chinese version of administered questionnaire. (DOCX 27 kb) Additional file 2: Survey-English.docx: English version of administered questionnaire. (DOCX 25 kb)

## Abbreviations

ASD: autism spectrum disorder
TCM: traditional Chinese medicine
