# Supplementary material for: Knowledge, attitudes, and perceptions of autism spectrum disorder in a stratified sampling of preschool teachers in China
Source: BMC Psychiatry. 2016 May 13;16:142. doi: 10.1186/s12888-016-0845-2 (PMC4865992; doi:10.1186/s12888-016-0845-2)
Supplement: Additional file 1: — Survey-Chinese.docx: Chinese version of administered questionnaire. (DOCX 27 kb) [file 12888_2016_845_MOESM1_ESM.docx]

**调查同意书**

老师：您好！

我们是中山大学公共卫生学院流行病与统计学系的师生。为了能更好地帮助患有孤独症（自闭症）的儿童，我们拟对广州幼儿园老师进行调查。本调查表主要想了解您对孤独症（自闭症）的了解程度和态度，只需花费您10分钟时间。该调查已获得中山大学公共卫生学院伦理委员会的批准（批件号L2016-045），调查形式为匿名调查，不会泄露您的隐私，请放心填写。为了保证问卷的有效性，请您尽量根据您的实际情况填写。如果您对本次调查有任何意见和建议，可与我们联系。联系电话：159-0201-4885。非常感谢您的积极配合和参与！

参加者编号 _ _ _ _ _ _ _ _

**一、个人基本信息 （请在适合您的答案前面的数字上打圈或在空白处填写。）**

1. 您的性别： （1）男 （2）女
2. 您的年龄：______（岁）

3. 您受教育程度水平：

（1）初中及以下 （2）高中 （3）中专 （4）大专 （5）大学本科（6）研究生及以上

4. 您在幼儿园工作的时间：____（年）

5. 您有无接受过儿童早期教育方面的专业训练: （1）有 （2）没有

6. 您有无接受过关于儿童特殊疾病（包括孤独症，多动症，其它发育失常的病症等）的训练:

（1）有 （2）没有

7. 在您的教学经历中，有无接触过患有上述疾病的儿童： （1）有 （2）没有

8. 您是否听说过孤独症（自闭症）？ （1）是 （2）否

**二、 有关儿童生长发育知识的问题：关于儿童的生长发育，下面列出了各种说法，请您判断该说法是否正确，并在相应的选项上画圈。**

| 1. | 三岁的儿童走路不稳是正常的。 | 对 错 不知道 |
| --- | --- | --- |
| 2. | 一岁半的儿童就确定了左右手偏好是正常的。 | 对 错 不知道 |
| 3. | 儿童直到四岁才会有虚拟想象，才能玩虚拟游戏。 | 对 错 不知道 |
| 4. | 三岁的儿童应该知道如何与小朋友轮流玩玩具。 | 对 错 不知道 |
| 5. | 五岁的儿童应该可以聊谈自己的日常活动。 | 对 错 不知道 |
| 6. | 如果儿童表现出注意力不集中，他可能有心理问题。 | 对 错 不知道 |
| 7. | 三岁的儿童听不懂简单的指令，例如听不懂 “站在门边”，“关电视”，“开灯”等指令，那是正常的。 | 对 错 不知道 |
| 8. | 男孩直到两岁才会说话是正常的。 | 对 错 不知道 |
| 9. | 三岁的儿童仍频繁的把物品放进嘴里是正常的。 | 对 错 不知道 |
| 10. | 儿童积极参加幼儿班组织的唱歌、跳舞等娱乐活动是有意义的。 | 对 错 不知道 |
| 11. | 如果一个三岁的儿童认识所有数字和英文字母，但不会说完整的句子，我们不用担心。 | 对 错 不知道 |
| 12. | 大部分儿童在五岁前都会有点多动和注意力不集中。 | 对 错 不知道 |
| 13. | 语言表达能力差的儿童，可能会更容易出现多动和注意力不集中。 | 对 错 不知道 |
| 14. | 有学习或行为问题的儿童可能存在潜在的家庭问题。 | 对 错 不知道 |
| 15. | 所有语言发育延迟的儿童均需要做听力检查。 | 对 错 不知道 |

**三、有关孤独症或自闭症知识的问题：下面列出了各种关于孤独症或自闭症的说法，请你判断该说法是否正确，并在相应的选项上画圈。**

| 1. | 孤独症儿童在两到三岁时就会表现出语言发育延迟。 | 对 错 不知道 |
| --- | --- | --- |
| 2. | 如果能够早期诊断并采用恰当的治疗方法，孤独症是可以被治愈的。 | 对 错 不知道 |
| 3. | 孤独症是由于父母对孩子的照顾不周导致的。 | 对 错 不知道 |
| 4. | 患有孤独症的儿童将来都考不上大学。 | 对 错 不知道 |
| 5. | 调整饮食可以有效改善孤独症儿童的症状。 | 对 错 不知道 |
| 6. | 孤独症儿童的视觉输入比听觉输入好，表现为看着学比听着学要好。 | 对 错 不知道 |
| 7. | 孤独症是一种发育障碍。 | 对 错 不知道 |
| 8. | 孤独症是一种心理疾病。 | 对 错 不知道 |
| 9. | 孤独症的患病率低于10%。 | 对 错 不知道 |
| 10. | 孤独症儿童没有社会依恋，甚至对父母也没有依恋。 | 对 错 不知道 |
| 11. | 自闭症的儿童长大后很可能得精神分裂症。 | 对 错 不知道 |
| 12. | 学校应该为自闭症患儿提供特殊教育，这非常重要。 | 对 错 不知道 |
| 13. | 自闭症儿童是故意捣乱，不听话。 | 对 错 不知道 |
| 14. | 父母社会经济地位和受教育水平越高，儿童越容易患上孤独症（自闭症）。 | 对 错 不知道 |
| 15. | 如果采用的治疗方法得当，大部分孤独症儿童可以最终康复。 | 对 错 不知道 |
| 16. | 自闭症的儿童不会有爱的行为。 | 对 错 不知道 |
| 17. | 孤独症是是阴阳失衡导致的。 | 对 错 不知道 |
| 18. | 孤独症的症状之一是身体的某个部位疼痛。 | 对 错 不知道 |

**四、您的态度：下面列出了一系列说法，根据您是否同意该说法在相应的选项上画圈。1 是坚决**

**不同意，6是坚决同意。**

| 1. | 有特殊需要的儿童应该进入主流学校接受正规教育。 | 1 2 3 4 5 6 |
| --- | --- | --- |
| 2. | 所有的学前班都应该接收这类有特殊需求的孩子。 | 1 2 3 4 5 6 |
| 3. | 对这类有特殊需求的孩子，学前班应该允许孩子的家长留在教室里看好自己的孩子。 | 1 2 3 4 5 6 |
| 4. | 所有的学前班都应该配备特殊教育的老师（或治疗师），从而能为这类有特殊需求的孩子提供特殊教育。 | 1 2 3 4 5 6 |
| 5. | 政府应该为特殊教育项目提供资金，让学校和学前教育机构能聘用受过特殊教育训练的老师，从而能为这类有特殊需求的孩子提供合适的教育服务。 | 1 2 3 4 5 6 |
| 6. | 有特殊需求孩子的家长也应该为这类特殊教育承担一定的费用。 | 1 2 3 4 5 6 |
| 7. | 中国现在已经提供了足够的特殊教育服务来满足这些有特殊需求的孩子。 | 1 2 3 4 5 6 |
| 8. | 政府应该为有特殊需求的孩子提供更多的服务，以满足他们的特殊需要。 | 1 2 3 4 5 6 |
| 9. | 保险政策应该被修订，把这些发育障碍疾病当做慢性疾病纳入医疗保险的服务范畴中。 | 1 2 3 4 5 6 |

**五、您对特殊教育具体实践的看法: 下面列出了一系列说法，根据您是否同意该说法在相应的选项上画圈。1 是坚决不同意，6是坚决同意。**

| 1. | 我觉得我能为这类有特殊需求的孩子提供帮助。 | 1 2 3 4 5 6 |
| --- | --- | --- |
| 2. | 我有兴趣参加儿童发育和行为障碍方面的培训。 | 1 2 3 4 5 6 |
| 3. | 如果接受了足够的培训，我愿意帮助班上有特殊需求的孩子。 | 1 2 3 4 5 6 |
| 4. | 我愿意为有特殊需求的儿童提供其它服务，比如生产制作适合于这类有特殊需求的孩子的教育材料、医疗服务等。 | 1 2 3 4 5 6 |
| 5. | 我很高兴让孩子的家长或治疗师留在班上帮助照顾孩子。学前教育里应该有特殊教育的老师。 | 1 2 3 4 5 6 |
| 6. | 我觉得学校有必要在教室和教学等方面为这类有特殊需求的孩子做出一些改善。 | 1 2 3 4 5 6 |
| 7. | 我愿意努力改善有特殊需求孩子的受教育现状。 | 1 2 3 4 5 6 |
| 8. | 我觉得我能够改善有特殊需求孩子的受教育现状。 | 1 2 3 4 5 6 |
| 9. | 这类有特殊需求孩子的家长有责任去为孩子们争取获得相应的服务。 | 1 2 3 4 5 6 |

**六、您听说过下列这些组织吗?**

| 1. | 北京星星雨教育研究所 | 有 没有 |
| --- | --- | --- |
| 2. | MoreToBaby关心孩子国际组织 | 有 没有 |
| 3. | AutismSpeaks自闭症国际组织 | 有 没有 |
| 4. | Kangna School广州市康纳学校 | 有 没有 |
| 5. | Shenzhen Autism Association深圳自闭症协会 | 有 没有 |
| 6. | NewHope Biomedical Centre新希望生物医学中心 | 有 没有 |
| 7. | 太阳船 | 有 没有 |

**七、您听说过下列这些治疗方法吗?**

| 1. | 应用行为分析法 | 有 没有 |
| --- | --- | --- |
| 2. | 结构化训练 | 有 没有 |
| 3. | 关系发展干预 | 有 没有 |
| 4. | 感觉统合训练 | 有 没有 |
| 5. | 听觉统合训练 | 有 没有 |

您已经完成了这个调查。 谢谢您的合作！

调查员：

调查时间：

调查地点（学校）：
