# Supplementary material for: Knowledge, attitudes, and perceptions of autism spectrum disorder in a stratified sampling of preschool teachers in China
Source: BMC Psychiatry. 2016 May 13;16:142. doi: 10.1186/s12888-016-0845-2 (PMC4865992; doi:10.1186/s12888-016-0845-2)
Supplement: Additional file 2: — Survey-English.docx: English version of administered questionnaire. (DOCX 25 kb) [file 12888_2016_845_MOESM2_ESM.docx]

**Survey Consent Form**

Teachers: Hello!

We are Sun Yat-Sen University School of Public Health epidemiology and biostatistics students. In order to better understand children with autism, we are surveying pre-school teachers in Guangzhou. The survey involves answering some general demographics questions and some questions about your knowledge and attitude towards autism. The survey takes about *10* minutes to complete. The purpose of the survey is to help assess the level of autism awareness currently in China. Your participation is completely voluntary, and your responses will be completely anonymous. The data I collect will be analyzed at the group level only. You do not have to answer any question you would rather not answer. There are no consequences if you decide not to complete the survey.

If you agree to complete the survey, please do NOT write your name on it. After you finish filling it out, please put the survey in the envelope and then place the envelope in the box provided. By filling out the survey you are consenting to participate.

If you do not want to complete the survey, just return the blank form and envelope to me now.

If you have any questions about the assignment or your rights as a volunteer participant in this subject, you can reach me at 159-0201-4885. This project has been approved by the Institutional Review Board of the Sun Yat-sen University College of Public Health (reference number: L2016-045).

Please keep this letter for your records. Thank you for your participation.

Participant number _ _ _ _ _ _ _ _

**Basic information**

Gender: M F

Age (years):

<20 20-24 25-29 30-34 35-39 40-44 > 45

Highest educational level achieved:

Middle school High school College Masters PhD

Years in pre-school education: ____

Received formal training in early childhood education: Y N

Training in special-needs disorders: Y N

Working experience with special-needs children :

□ Previous such experience

□ Previously taught a special needs child

□ Special-needs child in present class

Have you heard of autism?

Yes No

**Questions regarding understanding normal development: In the series of statements below, please circle each statement as true, false, or do not know.**

| 1. | At 3 years old, it is acceptable for a child to be still unsteady when walking. | T F DNK |
| --- | --- | --- |
| 2. | It is normal for a 1.5-year-old child to have already developed definite hand preference. | T F DNK |
| 3. | A child only develops make-believe play at 4 years old. | T F DNK |
| 4. | A 3-year-old child should be learning to take turns at play. | T F DNK |
| 5. | A 5-year-old child should be able to exchange conversation about daily activities and experiences. | T F DNK |
| 6. | A child who appears inattentive may actually be having fits. | T F DNK |
| 7. | It is normal for a 3-year-old child not to understand simple instructions. | T F DNK |
| 8. | It is normal for a boy to start speaking at the age of 2. | T F DNK |
| 9. | It is normal for 3-year-old children to frequently mouth objects (put things in their mouth). | T F DNK |
| 10. | A child should participate meaningfully during Music and Movement Time in nursery class. | T F DNK |
| 11. | There is no cause for concern if a 3-year-old child can recognize all the letters of the alphabets and numbers, but does not speak in sentences. | T F DNK |
| 12. | Most children are slightly hyperactive and inattentive before the age of 5. | T F DNK |
| 13. | A child with poor language skills can appear hyperactive and inattentive. | T F DNK |
| 14. | Children who have learning or behavioral problems may have underlying family concerns. | T F DNK |
| 15. | All children with speech and language delay should have a hearing test done. | T F DNK |

**Questions regarding Autistic Spectrum Disorder: In the series of statements below, please circle each statement as true, false, or do not know.**

| 1. | A child with ASD often presents with speech and language delay between 2 and 3 years old. | T F DNK |
| --- | --- | --- |
| 2. | ASD is curable if diagnosed early and the appropriate intervention provided. | T F DNK |
| 3. | Autism is caused by poor parenting skills or poor home care. | T F DNK |
| 4. | All children with ASD will be unable to pursue further education at the university level. | T F DNK |
| 5. | Changing the diet of a child with ASD will make a difference to his outcome. | T F DNK |
| 6. | A child with ASD often does better with visual input than with auditory input. | T F DNK |
| 7. | Autism is a developmental disorder. | T F DNK |
| 8. | Autism is a psychological problem. | T F DNK |
| 9. | Autism occurs in less than 10% of the population. | T F DNK |
| 10. | Autistic children do not show social attachments, even to parents. | T F DNK |
| 11. | Autistic children usually grow up to be schizophrenic adults. | T F DNK |
| 12. | It is important that autistic children receive special education services at school. | T F DNK |
| 13. | Autistic children are deliberately negativistic and noncompliant. | T F DNK |
| 14. | Autism occurs more commonly among higher socioeconomic and educational levels. | T F DNK |
| 15. | With the proper treatment, most autistic children eventually outgrow autism. | T F DNK |
| 16. | Autistic children do not show affectionate behaviors. | T F DNK |
| 17. | Autism is caused by an imbalance between yin and yang. | T F DNK |
| 18. | Autism has manifestations in physical pain in certain parts of the body. | T F DNK |

**Attitudes: For each statement below, please circle the letter that best corresponds to your attitude about the statement. 1 is strongly disagree, 6 is strongly agree.**

| 1. | Special-needs children should be integrated into mainstream school | 1 2 3 4 5 6 |
| --- | --- | --- |
| 2. | All pre-schools should allow children requiring special education to attend their classes while awaiting placement | 1 2 3 4 5 6 |
| 3. | Pre-schools should allow the presence of parents in class for children with special needs. | 1 2 3 4 5 6 |
| 4. | All pre-schools should have special education teachers and therapists to provide services for special-needs children who are attending classes there. | 1 2 3 4 5 6 |
| 5. | Government funding should be made available to facilitate staff employment in pre-schools to meet the needs of these children. | 1 2 3 4 5 6 |
| 6. | The parents should help bear the cost of services within the pre-schools. | 1 2 3 4 5 6 |
| 7. | There is adequate provision of services of special-needs children in China. | 1 2 3 4 5 6 |
| 8. | The government should allocate more resources for the provision of services for special needs children. | 1 2 3 4 5 6 |
| 9. | Insurance policies should be amended to include coverage for developmental disorders as chronic disabilities. | 1 2 3 4 5 6 |

**Views on special needs education: For each statement below, please circle the letter that best corresponds to your attitude about the statement. 1 is strongly disagree, 6 is strongly agree.**

| 1. | I feel equipped to handle children with special needs. | 1 2 3 4 5 6 |
| --- | --- | --- |
| 2. | I am interested to attend training in the area of childhood developmental and behavioral disorders. | 1 2 3 4 5 6 |
| 3. | If adequately trained, I am willing to have children with special needs in my class. | 1 2 3 4 5 6 |
| 4. | I am keen to be a partner in their management, e.g. use of specific visual aids, medication | 1 2 3 4 5 6 |
| 5. | I am happy to have parents or therapists sit in as helpers. There should be special education teachers within the pre-school community. | 1 2 3 4 5 6 |
| 6. | I see the need to implement changes in the classroom set-up to accommodate their needs. | 1 2 3 4 5 6 |
| 7. | I want to make a difference in the education of children with special needs. | 1 2 3 4 5 6 |
| 8. | I feel I can make a difference in the education of children with special needs. | 1 2 3 4 5 6 |
| 9. | The parents are responsible for obtaining services for their own special-needs children. | 1 2 3 4 5 6 |

**Have you heard of any of these organizations before?**

| 1. | Beijing Stars and Rain | Yes No |
| --- | --- | --- |
| 2. | MoreToBaby | Yes No |
| 3. | AutismSpeaks | Yes No |
| 4. | Kangna School | Yes No |
| 5. | Shenzhen Autism Association | Yes No |
| 6. | NewHope Biomedical Centre | Yes No |
| 7. | Tai Yang Chuan | Yes No |

**Have you heard of any of these treatments before?**

| 1. | Applied behavioral analysis | Yes No |
| --- | --- | --- |
| 2. | Structured training | Yes No |
| 3. | Relationship development intervention | Yes No |
| 4. | Sensory integration therapy | Yes No |
| 5. | Auditory integration therapy | Yes No |

You have finished the survey. Thank you for your participation!

Survey administer:

Administered time:

Administered location (school):
